# Single Nucleotide Polymorphisms of TCF7L2 Are Linked to Diabetic Coronary Atherosclerosis

**DOI:** 10.1371/journal.pone.0017978

**Published:** 2011-03-15

**Authors:** Axel Muendlein, Christoph H. Saely, Simone Geller-Rhomberg, Gudrun Sonderegger, Philipp Rein, Thomas Winder, Stefan Beer, Alexander Vonbank, Heinz Drexel

**Affiliations:** 1 Vorarlberg Institute for Vascular Investigation and Treatment, Feldkirch, Austria; 2 Private University of the Principality of Liechtenstein, Triesen, Principality of Liechtenstein; 3 Department of Medicine and Cardiology, Academic Teaching Hospital Feldkirch, Feldkirch, Austria; 4 Drexel University College of Medicine, Philadelphia, Pennsylvania, United States of America; University of Tor Vergata, Italy

## Abstract

**Background:**

Coronary artery disease (CAD) shares common risk factors with type 2 diabetes (T2DM). Variations in the transcription factor 7-like 2 (TCF7L2) gene, particularly rs7903146, increase T2DM risk. Potential links between genetic variants of the TCF7L2 locus and coronary atherosclerosis are uncertain. We therefore investigated the association between TCF7L2 polymorphisms and angiographically determined CAD in diabetic and non-diabetic patients.

**Methodology/Principal Findings:**

We genotyped TCF7L2 variants rs7903146, rs12255372, and rs11196205 in a cross-sectional study including 1,650 consecutive patients undergoing coronary angiography for the evaluation of established or suspected stable CAD. Significant CAD was diagnosed in the presence of coronary stenoses ≥50%. Variant rs7903146 in the total study cohort was significantly associated with significant CAD (adjusted additive OR = 1.29 [1.09–1.53]; p = 0.003). This association was strong and significant in T2DM patients (n = 393; OR = 1.91 [1.32–2.75]; p = 0.001) but not in non-diabetic subjects (OR = 1.09 [0.90–1.33]; p = 0.370). The interaction risk allele by T2DM was significant (p_interaction_ = 0.002), indicating a significantly stronger impact of the polymorphism on CAD in T2DM patients than in non-diabetic subjects. TCF7L2 polymorphisms rs12255372 and rs11196205 were also significantly associated with CAD in diabetic patients (adjusted additive OR = 1.90 [1.31–2.74]; p = 0.001 and OR = 1.75 [1.22–2.50]; p = 0.002, respectively). Further, haplotype analysis demonstrated that haplotypes including the rare alleles of all investigated variants were significantly associated with CAD in the whole cohort as well as in diabetic subjects (OR = 1.22 [1.04–1.43]; p = 0.013 and OR = 1.67 [1.19–2.22]; p = 0.003, respectively).

**Conclusions/Significance:**

These results suggest that TCF7L2 variants rs7903146 rs12255372, and rs11196205 are significantly associated with angiographically diagnosed CAD, specifically in patients with T2DM. TCF7L2 therefore appears as a genetic link between diabetes and atherosclerosis.

## Introduction

Type 2 diabetes (T2DM) confers a two- to threefold increased risk of coronary artery disease (CAD) [1], [2]. Importantly, T2DM and CAD share common risk factors and both diseases show a strong genetic background [3]–[5]. Recently, transcription factor 7-like 2 (TCF7L2), located on chromosome 10q25.3, has been identified as a major T2DM susceptibility gene [6]. Single nucleotide polymorphisms (SNPs) of TCF7L2 have been consistently associated with T2DM in populations of different ethnic descent, making TCF7L2 one of the most important locus known today to predispose for T2DM [7]–[9]. Among noted SNPs, variant rs7903146 was found to be most significantly associated with T2DM risk [6]–[9]. Further, TCF7L2 SNPs rs12255372 and rs11196205 have been linked with impaired glucose metabolism [8]–[10] and an increased diabetes risk. [6], [8], [9].

Several studies have shown that TCF7L2 influences the risk of T2DM via impairment of beta-cell function (BCF) [11], [12]. However, the exact mechanisms through which TCF7L2 affects the susceptibility to T2DM remain unclear [13], [14]. The gene TCF7L2 encodes the transcription factor TCF-4 [15], which serves as a nuclear receptor for beta-catenin in the Wingless-type (Wnt) signalling pathway [16]. The beta-catenin/TCF-4 transcriptional complex is involved in a variety of biological events. In particular, it has been shown to play an important role in vascular remodelling through the regulation of smooth muscle cell proliferation and endothelial cell growth [17]–[19]. TCF7L2 may therefore contribute not only to the development of T2DM but also to the development of CAD. However potential links between genetic variants of the TCF7L2 locus and CAD are uncertain [20], [21].

We therefore aimed at investigating the association of the TCF7L2 SNPs rs7903146, rs12255372, and rs11196205 with coronary atherosclerosis in a large cohort of well characterized consecutive patients undergoing coronary angiography. In particular, we investigated whether the association between TCF7L2 polymorphisms and angiographically characterized CAD differs between patients with diabetes and non-diabetic individuals.

## Results

### Characteristics of the study population

Overall, the characteristics of our patients were typical for patients undergoing coronary angiography for the evaluation of CAD, with a preponderance of male gender (66.4%) and a high prevalence of T2DM (23.8%), hypertension (53.5%), and smoking (59.1%). Coronary angiography revealed significant coronary stenoses in 57.6% of the patients; 58.3% of our patients with diabetes were on anti-diabetic medication. Clinical and biochemical characteristics according to the presence of T2DM are summarized in table 1.

**Table 1:** Clinical and biochemical subject characteristics with respect to type 2 diabetes mellitus.

	No T2DM	T2DM	P value
Individuals (n)	1257	393	-
Age (years)	63.7±10.8	65.4±9.9	0.001
Male sex (%)	66.2	67.2	0.718
BMI (kg/m^2^)	27.1±4.1	29.1±4.8	<0.001
Hypertension (%)	52.1	58.0	0.041
Smoking (%)	57.0	65.6	0.002
Total cholesterol (mg/dl)	208±45	192±47	<0.001
LDL cholesterol (mg/dl)	132±38	118±38	<0.001
HDL cholesterol (mg/dl)	55±17	49±14	<0.001
Triglycerides (mg/dl)	142±90	171±112	<0.001
Use of statins (%)	43.4	54.7	<0.001
Insulin (µU/ml)	10.3±9.0	18.7±45.6	<0.001
Glucose (mmol/l)	5.4±0.7	8.4±2.7	<0.001
HOMA IR	2.6±2.8	6.3±6.9	<0.001
HOMA BCF	116±89	86±87	<0.001

Differences in categorical variables were tested for statistical significance with the Chi-square test. For continuous variables t-tests were applied. Non-normally distributed variables [i.e. age, BMI, HDL cholesterol, LDL cholesterol, triglycerides, fasting insulin, fasting glucose, homeostasis model assessment (HOMA) insulin resistance (IR), and HOMA beta cell function (BCF)] were log-transformed prior to statistical analysis. Continuous variables are given as mean ± SD (of non log-transformed values).

### Genoytping analysis

Genotypes were successfully called in 1,650 patients for SNP rs7903146 (100%), in 1,645 patients for SNP rs12255372 (99.7%), and in 1,644 patients for SNP rs11196205 (99.6%). Results of re-genotyping analysis of 92 randomly selected samples were 100% in agreement with the initial genotyping results and all three TCF7L2 SNP genotype frequencies did not deviate significantly from Hardy-Weinberg equilibrium (respective Chi-squared values as well as p-values are given in table S1). These genotyping quality controls all indicate a high genotyping data quality. Further, the analysed TCF7L2 SNPs were in linkage disequilibrium: Pairwise squared correlation coefficients were r^2^ = 0.747 between rs7903146 and rs12255372, r^2^ = 0.491 between rs7903146 and rs11196205, and r^2^ = 0.461 between rs12255372 and rs11196205.

### Association of TCF7L2 variants with clinical and biochemical subject characteristics

Associations between TCF7L2 variants rs7903146, rs12255372, and rs11196205 and subject characteristics in the total cohort, as well as in patients with T2DM and in non-diabetic subjects are presented in tables S2, S3, and S4, respectively. Variants rs7903146 and rs12255372 were significantly associated with increased glucose levels, as well with a reduced HOMA index of beta cell function in the total cohort. Further, variants rs7903146 and rs12255372 were significantly associated with T2DM in our study population (additive OR = 1.33 [1.13–1.58]; p = 0.001, and OR = 1.36 [1.15–1.61]; p<0.001, respectively). Also, for both variants diabetes duration significantly increased from the homozygous genotype of the common allele, over the heterozygous genotype, to the homozygous genotype of the rare allele (7.7±8.6, 8.1±7.3, and 9.2±6.8 years, p = 0.024 for variant rs7903146 and 7.5±8.4, 8.5±7.5, and 9.0±6.6 years, p = 0.045 for variant rs12255372). Variant rs11196205 was neither associated with T2DM nor with diabetes duration (p = 0.058 and p = 0.547, respectively).

### Association of TCF7L2 variants with angiographically characterized CAD

Table 2 presents the prevalence of significant CAD according to the genotypes of variants rs7903146, rs12255372, and rs11196205, as well as allelic ORs from unadjusted and multivariable logistic regression analysis. Regression models were built in a stepwise manner: Model 1 is unadjusted, model 2 adjusts for age, gender, BMI, smoking, hypertension, serum levels of HDL cholesterol, LDL cholesterol, as well as for statin use, and model 3 for the covariates included in model 2 and, additionally, for serum insulin and plasma glucose as well as for anti-diabetic medication and haemoglobin A1c (HbA1c) levels. In all regression models, variant rs7903146 proved significantly associated with angiographically characterized CAD in the total study population. The association between variant rs12255372 and CAD was not significant in unadjusted regression analysis, but became significant in regression models 2 and 3. SNP rs11196205 was not associated with CAD in any regression model.

**Table 2:** Association between TCF7L2 variants and significant coronary artery disease in the total cohort.

Variant	rs7903146			rs12255372			rs11196205		
Genotype	CC	CT	TT	GG	GT	TT	GG	GC	CC
Frequency CAD− (%)	50.9	39.6	9.6	53.4	38.0	8.6	29.4	50.1	20.6
Frequency CAD+ (%)	45.3	43.9	10.8	48.5	41.9	9.6	28.3	49.3	22.3
OR [95%CI]*	1.17 [1.01–1.36]			1.15 [0.99–1.34]			1.06 [0.92–1.21]		
p*	0.038			0.073			0.432		
OR [95%CI]†	1.29 [1.09–1.53]			1.27 [1.07–1.51]			1.10 [0.94–1.29]		
p†	0.003			0.006			0.223		
OR [95%CI] ‡	1.28 [1.07–1.54]			1.26 [1.04–1.51]			1.08 [0.91–1.29]		
p‡	0.008			0.017			0.359		

Additive odds ratios (OR), 95% confidence intervals (CI), and p-values were calculated by logistic regression analyses: * Unadjusted. † Adjusted for age, gender, BMI, smoking, hypertension, serum levels of HDL cholesterol, LDL cholesterol, as well as for statin use. ‡ Adjusted for the same covariates as in model (†) and additionally for serum insulin, plasma glucose, haemoglobin A1c, and anti-diabetic medication. CAD indicates coronary artery disease. T2DM indicates type 2 diabetes mellitus.

Subgroup analysis with respect to the presence of T2DM showed that all variants were significantly associated with CAD in patients with T2DM, while they did not significantly influence CAD risk in non-diabetic individuals. After further adjustment for the duration of T2DM, the association between variant rs7903146, rs12255372, and rs11196205 and CAD remained significant among patients with diabetes (OR = 2.34 [1.32–4.15]; p = 0.004, OR = 2.00 [1.34–3.00]; p = 0.001, and OR = 1.96 [1.32–2.91]; p = 0.001, respectively).

To test whether the difference in the atherogenicity of variant rs7903146 differed between patients with T2DM and non-diabetic subjects in a statistically significant manner, interaction terms were entered into logistic regression analyses (table 3). The interaction terms ‘T2DM by SNP’ (additive model) of all investigated TCF7L2 variants were significant indicating that TCF7L2 variants had a significantly stronger impact on CAD in patients with T2DM than in non-diabetic patients. T2DM thus significantly modulates the association of these TCF7L2 SNPs with angiographically characterized CAD.

**Table 3:** Association between TCF7L2 variants and significant coronary artery disease with respect to the presence of type 2 diabetes mellitus.

Variant	rs7903146						rs12255372						rs11196205					
Presence of T2DM	T2DM (−)			T2DM (+)			T2DM (−)			T2DM (+)			T2DM (−)			T2DM (+)		
Genotype	CC	CT	TT	CC	CT	TT	GG	GT	TT	GG	GT	TT	GG	GC	CC	GG	GC	CC
Frequency CAD− (%)	51.5	38.4	10.1	48.1	44.4	7.5	53.6	37.5	8.8	52.3	40.2	7.6	29.0	49.7	21.3	31.1	51.5	17.4
Frequency CAD+ (%)	48.1	43.3	8.6	37.7	45.4	16.9	51.7	41.0	7.3	40.0	44.3	15.8	31.1	48.0	20.9	21.2	52.7	26.2
OR [95%CI]*	1.05 [0.88–1.24]			1.54 [1.12–2.12]			1.01 [0.85–1.20]			1.57 [1.14–2.16]			0.95 [0.81–1.11]			1.49 [1.09–2.03]		
p*	0.611			0.007			0.934			0.006			0.532			0.012		
p_interaction_ T2DMxSNP*	0.034						0.017						0.012					
OR [95%CI]†	1.09 [0.90–1.33]			1.91 [1.32–2.75]			1.06 [0.87–1.30]			1.90 [1.31–2.74]			0.95 [0.79–1.13]			1.75 [1.22–2.50]		
p†	0.370			0.001			0.540			0.001			0.557			0.002		
p_interaction_ T2DMxSNP†	0.002						0.001						0.001					
OR [95%CI] ‡	1.09 [0.89–1.35]			2.02 [1.34–3.03]			1.06 [0.85–1.31]			2.01 [1.35–3.02]			0.91 [0.75–1.10]			1.98 [1.33–2.95]		
p‡	0.410			0.001			0.604			0.001			0.322			0.001		
p_interaction_ T2DMxSNP‡	0.003						0.002						<0.001					

Additive odds ratios (OR), 95% confidence intervals (CI), and p-values, as well as p_interaction_-values were calculated by logistic regression analyses: * Unadjusted. † Adjusted for age, gender, BMI, smoking, hypertension, serum levels of HDL cholesterol, LDL cholesterol, as well as for statin use. ‡ Adjusted for the same covariates as in model (†) and additionally for serum insulin, plasma glucose, haemoglobin A1c, and anti-diabetic medication. CAD indicates coronary artery disease. T2DM indicates type 2 diabetes mellitus. SNP indicates single nucleotide polymorphism.

To further investigate the effect of elevated glucose levels on the association between these variants and the presence of significant coronary lesions we performed a subgroup analysis with respect to subjects with impaired fasting glucose (n = 448). Variants rs7903146, rs12255372, and rs11196205 were not significantly associated with CAD in this subgroup (OR = 0.85 [0.63–1.13]; p = 0.258, OR = 0.75 [0.56–1.01]; p = 0.057, and OR = 0.86 [0.66–1.12], 0.258, respectively).

To evaluate the quantitative contribution of TCF7L2 SNPs to angiographically characterized coronary atherosclerosis in our patients, we investigated the association of these genetic variants with the extent of coronary lesions, defined as the mean number of significant coronary stenoses in a given patient (table 4). Variant rs7903146 and rs12255372 were significantly associated with the extent of CAD in patients with T2DM, while they did not significantly influence the extent of CAD in non-diabetic individuals or in the total study cohort. SNP rs11196205 was associated with the extent of CAD in neither study subgroup.

**Table 4:** Association between TCF7L2 variants and the extent of coronary artery disease.

Variant	Genotype	All patients		Non-diabetic patients		Diabetic patients	
		Extent (±SD)	p-value	Extent (±SD)	p-value	Extent (±SD)	p-value
rs7903146	CC	1.32 ± 1.58		1.27 ± 1.54		1.50 ± 1.71	
	CT	1.46 ± 1.66	0.053	1.38 ± 1.63	0.963	1.70 ± 1.70	0.043
	TT	1.26 ± 1.45		0.96 ± 1.17		1.93 ± 1.75	
rs12255372	GG	1.34 ± 1.58		1.30 ± 1.57		1.49 ± 1.65	
	GT	1.44 ± 1.65	0.321	1.33 ± 1.60	0.673	1.77 ± 1.77	0.047
	TT	1.28 ± 1.46		1.00 ± 1.24		1.84 ± 1.69	
rs11196205	GG	1.38 ± 1.60		1.36 ± 1.57		1.46 ± 1.72	
	GC	1.38 ± 1.62	0.983	1.30 ± 1.63	0.209	1.63 ± 1.60	0.058
	CC	1.35 ± 1.55		1.56 ± 1.35		1.91 ± 1.92	

The extent of coronary artery disease was calculated as the number of significant coronary stenoses in a given patient. SD indicates standard deviation. P-values were calculated by the Jonckheere-Terpstra-test.

### Association of TCF7L2 haplotypes with angiographically characterized CAD

Estimated frequencies of the five most common haplotypes and their associations with CAD with respect to the presence of T2DM are shown in table 5. The two frequent haplotype blocks including either the common or the rare alleles of variants rs7903146, rs12255372, and rs11196205 were significantly associated with angiographically determined CAD in patients with T2DM. A third common haplotype block carrying the common alleles of variants rs7903146 and rs12255372, but the rare allele of variant rs11196205 was not associated with the presence of CAD. Also, remaining (less common) haplotypes were not associated with significant coronary lesions. Neither haplotype in non-diabetic subjects was associated with CAD. In the whole cohort, the haplotype block including the rare alleles of TCF7L2 variants showed significant association with CAD (table S5).

**Table 5:** Estimated frequency of common haplotypes and their associations with significant coronary artery disease with respect to the presence of T2DM.

			Non-diabetic patients				Diabetic patients			
	Haplotype		CAD (−) %	CAD (+) %	OR [95%CI]	P value	CAD (−) %	CAD (+) %	OR [95%CI]	P value
rs7903146 C>T	rs12255372 G>T	rs11196205 G>C								
C	G	G	53.1	54.6	1.06 [0.91–1.24]	0.468	55.6	45.8	0.67 [0.50–0.90]	0.009
C	G	C	15.6	13.7	0.86 [0.69–1.08]	0.193	11.8	12.0	1.03 [0.65–1.62]	0.908
C	T	C	1.9	1.0	0.53 [0.26–1.01]	0.050	2.8	2.4	0.86 [0.34–2.21]	0.755
T	G	C	3.5	3.5	1.01 [0.65–1.54]	0.983	4.6	3.0	0.66 [0.31–1.42]	0.286
T	T	C	25.2	26.7	1.08 [0.90–1.29]	0.410	24.3	35.1	1.67 [1.19–2.22]	0.003

ORs (odds ratios), 95%CIs (confidence intervals), and p values were calculated by chi-squared tests by comparing each haplotype to all remaining haplotypes. CAD indicates coronary artery disease.

## Discussion

In the present work, we report a positive association of the TCF7L2 variants rs7903146, rs12255372, and rs11196205 with coronary atherosclerosis, particularly in patients with T2DM.

Further, the results from our investigation are well in line with numerous other studies consistently showing that TCF7L2 variants rs7903146 and rs12255372 are associated with T2DM [6]–[9] and influence the risk of diabetes via pancreatic beta cell dysfunction [11], [12]. Variant rs11196205 was not significantly associated with T2DM. Also other studies found a weaker association between diabetes and this variant compared to variant rs7903146 and rs12255372, respectively [6], [9]. However, variant rs11196205 was significantly associated with CAD in diabetic patients in our study. Further, haplotype analysis demonstrated that haplotypes including the rare alleles of all investigated variants were significantly associated with CAD in the whole cohort as well as in diabetic subjects surpassing the effect of the individual SNPs. This observation points to a combined effect of investigated SNPs on the cardiovascular risk but also indicates that other SNPs located in this haplotype may contribute to the development of CAD.

Of note, recently the Atherosclerosis Risk in Communities (ARIC) Study did not find significant associations between TCF7L2 SNPs and incident vascular events [20].

However, in contrast to vascular events, which constitute the last step of atherothrombotic CAD and eventually are precipitated by thrombogenic factors, coronary angiography preferentially assesses atherosclerosis. Our data therefore indicate a role of TCF7L2 in atherogenesis.

The TCF7L2 encoded protein TCF-4 plays a distinct role in the Wnt signalling pathway [16], which has been shown to take part in vascular remodelling by the regulation of smooth muscle cell proliferation [17], [19] and of endothelial cell growth [18]. Moreover, a crosstalk between the Wnt signalling pathway and the nuclear factor-kappaB pathway has been proposed [22], [23], which regulates the expression of several inflammatory genes implicated in atherosclerosis [24]. Of note, TCF7L2 variants are associated with elevated blood glucose. Elevated glucose concentrations per se have been shown to activate nuclear factor κ-B. [25], [26]. It therefore can be hypothesized that TCF7L2 may influence the development of atherosclerosis i) by direct regulation of smooth muscle cell proliferation and ii) by turning on nuclear factor-kappaB pathway either directly through the Wnt signalling pathway, or indirectly through increased blood glucose levels (via impairment of beta cell function), or both, directly and indirectly.

Variants rs7903146, rs12255372, and rs11196205 in our study were associated with significant CAD in particular in diabetic individuals. It therefore can be further speculated that hyperglycaemia, which in turn predisposes to a proinflammatory and proatherogenic state is essential for the adverse impact of TCF7L2 on atherogenesis. However, even after adjustment for glucose and HbA1c levels associations between TCF7L2 variants and CAD remained significant in diabetic subjects. Therefore, relative hyperglycaemia among patients with known T2DM does not appear to explain the observed association. Another explanation could be that, because subjects carrying TCF7L2 diabetic risk-alleles are more likely to have progression from impaired glucose tolerance to T2DM [8], the duration of exposure of T2DM could be longer in risk-allele carriers compared to diabetic patients with the wild-type genotype, promoting the progress of atherogenesis. Indeed, in our study polymorphisms rs7903146 and rs12255372 were significantly associated with diabetes duration. This relation may contribute to the increased risk of CAD among T2DM risk-allele carriers but does not fully explain it: The variants in our investigation remained associated with CAD after adjustment for diabetes duration.

T2DM and CAD share common risk factors, but genes associated with both conditions are rare [27], [28]. We found that TCF7L2 variants are associated with both T2DM and angiographically diagnosed CAD, and that there are significant interactions between diabetes status and atherogenicity. These observations may point to a genetic link between diabetogenicity and atherogenicity mediated by the TCF7L2 gene.

In contrast to our findings, a recent study by Sousa et al. [21] found that the T-allele of TCF7L2 variant rs7903146 was associated with a higher prevalence and severity of coronary atherosclerosis in non-diabetic patients, but not in patients with T2DM. From our data we cannot provide a definite explanation for this discrepancy. It should be noted that there are some differences between the two studies concerning subject characteristics (e.g. mean age, prevalence of male sex and hypertension, as well as plasma levels of cholesterol) and data analysis (e.g. additive vs. dominant genetic model of inheritance), respectively, which may contribute to the divergent findings. In addition, the patients for the study by Sousa et al were recruited in Brazil and our patients in Austria, Europe, which of course implies ethnical differences. Whatsoever, our study provides approximately twice the sample-size of the study by Sousa et al. [21] (1,650 vs. 896 patients) and firmly establishes our results at least for a white European population.

Our study has strengths and limitations. An important strength and central feature of our study design is the angiographical characterisation of all subjects. By design, our study population of angiographied coronary patients is selected; our results therefore are not necessarily applicable to the general population. However, the high-risk patient population we chose to investigate is of particular clinical interest.

We conclude that TCF7L2 variants rs7903146, rs12255372, and rs11196205 are significantly associated with angiographically diagnosed CAD and that this association is significantly modulated by the presence of T2DM. TCF7L2, therefore, appears as an interesting candidate for a genetic link between atherogenesis and diabetogenesis.

## Materials and Methods

### Ethics Statement

The study has been carried out in accordance with the principles of the Declaration of Helsinki and the Ethics Committee of the Medical University of Innsbruck approved the present study; all participants gave written informed consent.

### Study subjects

We investigated a total of 1,661 white patients undergoing coronary angiography for the evaluation of suspected or established stable CAD at the Department of Medicine and Cardiology of the Academic Teaching Hospital Feldkirch, Austria. Ethnicity was ascertained by clinical adjudgement and family history. Patients with type 1 diabetes (n = 11) were excluded, thus data are reported for 1,650 individuals for the present study. Information on conventional cardiovascular risk factors such as a history of smoking, hypertension, or diabetes was obtained by a standardized interview. BMI was calculated as body weight (kg)/height (m)^2^. T2DM was diagnosed according to WHO criteria [29], and hypertension according to the Seventh Report of the Joint National Committee on Prevention, Detection, Evaluation, and Treatment of High Blood Pressure [30] was defined as arterial blood pressure greater than or equal to either 140 mm Hg systolic or 90 mm Hg diastolic. Diagnosis of impaired fasting glucose was made on the basis of fasting plasma glucose levels between 5.6 and 6.9 mmol/l [31]. Statin use was defined as drug intake of at least one month. Coronary angiography was performed with the Judkins technique [32]. Significant CAD was diagnosed in the presence of significant coronary stenoses with lumen narrowing of at least 50% and the extent of CAD was calculated as the number of significant coronary stenoses in a given patient, as described previously [33], [34].

### Measurement of biochemical variables

Venous blood samples were collected after an overnight fast of 12 hours before angiography was performed. The serum levels of triglycerides, total cholesterol, LDL cholesterol, and HDL cholesterol were determined by using enzymatic hydrolysis and precipitation techniques (Triglycerides GPO-PAP, CHOD/PAP, QuantolipLDL, QuantolipHDL; Roche, Basel, Switzerland) on a Hitachi-Analyzer 717 or 911. Fasting glucose levels were measured enzymatically from venous fluoride plasma by the hexokinase method (Roche) on a Hitachi 717 or 911. Fasting serum insulin was measured by an enzyme immunoassay on an AIA 1200 (Tosoh). HbA1c was determined by high-performance liquid chromatography on a Menarini-ArkrayKDKHA8140 (Arkray, Kyoto, Japan). We estimated BCF and insulin resistance (IR) from fasting plasma glucose and serum insulin using homeostasis model assessment (HOMA) [35]. HOMA BCF was calculated by using the formula: 20× fasting insulin (µU/ml)/[fasting glucose (mmol/l) −3.5]. HOMA IR was calculated by using the formula: [fasting insulin (µU/ml) × fasting glucose (mmol/l)]/22.5.

### Genotyping

Genomic DNA was extracted from EDTA blood or clotted blood samples using the peqGOLD® Blood DNA Mini kit (PEQLAB Biotechnologie Ltd., Erlangen, Germany). Genotyping of TCF7L2 variants rs7903146, rs12255372, and rs11196205 were carried out by the 5′ nuclease assay using TaqMan® MGB probes on a LightCycler® 480 Real-Time PCR System (F. Hoffmann-La Roche Ltd, Basel, Switzerland). TaqMan® MGB probes were provided together with corresponding PCR primers by the Assay-on-demand™ service (Applied Biosystems, Forster City, CA). The 5′ nuclease assay was performed in a 6 µl volume, comprising 10–30 ng genomic DNA, 1× TaqMan® Universal PCR Master Mix (Applied Biosystems), and 1× primer/probe mix under the following amplification conditions: 10 min at 95°C and 40 cycles at 92°C for 15 s and 60°C for 1 min. Reference controls as well as non template controls were included in each run. Genotypes were automatically determined by LightCycler®software 1.5 followed by a visual control of accurate genotype classification. Further, genotyping of 92 randomly selected samples was replicated to assess quality of SNP genotyping.

### Statistical analysis

Differences in categorical variables were tested for statistical significance with the Chi-square test. For continuous variables the t-test and ANOVA were applied. The Kolmogorov-Smirnov test was used to test, if continuous variables are normally distributed. In addition, a normal quantile-quantile plot of residuals of each variable was generated and visually inspected to test for normality. Non-normally distributed variables (i.e. age, BMI, LDL cholesterol, HDL cholesterol, triglycerides, fasting insulin, fasting glucose, HbA1c, HOMA IR, and HOMA BCF, as well as T2DM duration) were log-transformed prior to statistical analysis. Further, logistic regression analyses were performed for evaluating the association of the TCF7L2 variant with angiographically characterized CAD. To evaluate the association of the TCF7L2 SNP with the extent of coronary lesions the Jonckheere-Terpstra-test was applied. These statistical analyses were performed with the software package SPSS 11.0 for Windows (SPSS, Inc., Chicago, IL). Observed numbers of each genotype were compared with those expected to test whether the sample was in Hardy-Weinberg equilibrium using the Chi-Square test with one degree of freedom. To measure linkage disequilibrium, the squared correlation coefficient r^2^ was calculated for each pair of SNPs using CubeX software (http://www.oege.org/software/cubex [36]). Haplotype frequencies were evaluated by the Estimate Haplotype (EH) program (ftp://linkage.rockefeller.edu/software/eh [37]). The distribution of continuous variables is given as mean ± SD. (of non log-transformed values). Statistical significance was defined as a two-tailed p value <0.05.

For power calculations the software package Quanto 1.2.3 was used [38]. Power analysis was calculated for an additive model of inheritance. On the basis of a minor allele frequency of 30% on average of variants rs7903146 and rs12255372, and 46% of variant rs11196205, respectively, reported in the literature for a European population [7], [8], [10] and assuming a prevalence of significant coronary stenoses of 60%, *a priori* power analysis indicated that 1,500 patients would be sufficient to demonstrate an OR of 1.25 for rs7903146 and rs12255372 and an OR of 1.23 for rs11196205, at an alpha fault of 0.05 with a power of 80%. Under the same parameter settings 380 patients would be needed to demonstrate an OR of 1.55 for rs7903146 and rs12255372 and an OR of 1.50 for rs11196205, at an alpha fault of 0.05 with a power of 80%.

## Supporting Information

Table S1 **Testing for Hardy-Weinberg equilibrium.** *X* ^2^ refers to chi-squared.
(DOC)

Table S2 **Subject characteristics with respect to genotypes of rs7903146.** Differences in categorical variables were tested for statistical significance with the Chi-square test. For continuous variables ANOVA was applied. Non- normally distributed variables [i.e. age, BMI, HDL cholesterol, LDL cholesterol, triglycerides, fasting insulin, fasting glucose, homeostasis model assessment (HOMA) insulin resistance (IR), HOMA beta cell function (BCF), and haemoglobin A1c (HbA1c)] were log-transformed prior to statistical analysis. Continuous variables are given as mean ± SD (of non log-transformed values).
(DOC)

Table S3 **Subject characteristics with respect to genotypes of rs12255372.** Differences in categorical variables were tested for statistical significance with the Chi-square test. For continuous variables ANOVA was applied. Non- normally distributed variables [i.e. age, BMI, HDL cholesterol, LDL cholesterol, triglycerides, fasting insulin, fasting glucose, homeostasis model assessment (HOMA) insulin resistance (IR), HOMA beta cell function (BCF), and haemoglobin A1c (HbA1c)] were log-transformed prior to statistical analysis. Continuous variables are given as mean ± SD (of non log-transformed values).
(DOC)

Table S4 **Subject characteristics with respect to genotypes of rs11196205.** Differences in categorical variables study were tested for statistical significance with the Chi-square test. For continuous variables ANOVA was applied. Non- normally distributed variables [i.e. age, BMI, HDL cholesterol, LDL cholesterol, triglycerides, fasting insulin, fasting glucose, homeostasis model assessment (HOMA) insulin resistance (IR), HOMA beta cell function (BCF), and haemoglobin A1c (HbA1c)] were log-transformed prior to statistical analysis. Continuous variables are given as mean ± SD (of non log-transformed values).
(DOC)

Table S5 **Estimated frequency of common haplotypes and their associations with significant coronary artery disease.** Odds ratios (OR), 95% confidence intervals (CI), and p-values were calculated by chi-squared tests by comparing each haplotype to all remaining haplotypes.
(DOC)
