# Supplementary material for: Single Nucleotide Polymorphisms of TCF7L2 Are Linked to Diabetic Coronary Atherosclerosis
Source: PLoS One. 2011 Mar 15;6(3):e17978. doi: 10.1371/journal.pone.0017978 (PMC3058059; doi:10.1371/journal.pone.0017978)
Supplement: Table S1 — Testing for Hardy-Weinberg equilibrium. X 2 refers to chi-squared. (DOC) [file pone.0017978.s001.doc]

|  | Total cohort | | No T2DM | | T2DM | |
| --- | --- | --- | --- | --- | --- | --- |
|  | *X*^2^ | p-value | *X*^2^ | p-value | *X*^2^ | p-value |
| rs7903146 | 0.84 | 0.359 | 0.36 | 0.549 | 0.26 | 0.610 |
| rs12255372 | 1.35 | 0.245 | 0.30 | 0.584 | 1.02 | 0.313 |
| rs11196205 | 0.01 | 0.920 | 0.32 | 0.572 | 0.83 | 0.362 |
