# Supplementary material for: Single Nucleotide Polymorphisms of TCF7L2 Are Linked to Diabetic Coronary Atherosclerosis
Source: PLoS One. 2011 Mar 15;6(3):e17978. doi: 10.1371/journal.pone.0017978 (PMC3058059; doi:10.1371/journal.pone.0017978)
Supplement: Table S2 — Subject characteristics with respect to genotypes of rs7903146. Differences in categorical variables were tested for statistical significance with the Chi-square test. For continuous variables ANOVA was applied. Non- normally distributed variables [i.e. age, BMI, HDL cholesterol, LDL cholesterol, triglycerides, fasting insulin, fasting glucose, homeostasis model assessment (HOMA) insulin resistance (IR), HOMA beta cell function (BCF), and haemoglobin A1c (HbA1c)] were log-transformed prior to statistical analysis. Continuous variables are given as mean ± SD (of non log-transformed values). (DOC) [file pone.0017978.s002.doc]

|  | Total cohort | | | | No T2DM | | | | T2DM | | | |
| --- | --- | --- | --- | --- | --- | --- | --- | --- | --- | --- | --- | --- |
| rs7903146 (C>T) | CC | CT | TT | P value | CC | CT | TT | P value | CC | CT | TT | P value |
| Individuals (n) | 786 | 694 | 170 | - | 624 | 517 | 116 | - | 162 | 177 | 54 | - |
| Age (years) | 63.6 ± 10.5 | 64.7 ± 10.6 | 64.1 ± 10.9 | 0.177 | 63.3 ± 10.7 | 64.3 ± 10.7 | 63.5 ± 11.5 | 0.319 | 64.9 ± 10.0 | 65.9 ± 10.0 | 65.6 ± 9.4 | 0.652 |
| Male sex (%) | 67.3 | 67.7 | 57.1 | 0.073 | 66.0 | 68.3 | 57.8 | 0.424 | 72.2 | 66.1 | 55.6 | 0.024 |
| BMI (kg/m^2^) | 27.7 ± 4.5 | 27.3 ± 4.1 | 27.9 ± 4.9 | 0.284 | 27.1 ± 4.1 | 26.9 ± 4.0 | 27.3 ± 4.2 | 0.575 | 29.7 ± 5.1 | 28.5 ± 4.1 | 29.1 ± 5.9 | 0.107 |
| Hypertension (%) | 52.5 | 55.5 | 50.3 | 0.867 | 50.8 | 54.7 | 47.3 | 0.842 | 41.0 | 42.4 | 43.4 | 0.728 |
| Smoking (%) | 59.8 | 59.8 | 52.9 | 0.227 | 57.2 | 58.0 | 51.7 | 0.528 | 69.8 | 65.0 | 55.6 | 0.062 |
| Total cholesterol (mg/dl) | 206 ± 46 | 203 ± 46 | 203 ± 47 | 0.520 | 209 ± 46 | 208 ± 45 | 207 ± 43 | 0.928 | 195 ± 46 | 188 ± 47 | 192 ± 53 | 0.433 |
| LDL cholesterol (mg/dl) | 130 ± 39 | 129 ± 39 | 125 ± 38 | 0.313 | 132 ± 39 | 133 ± 38 | 129 ± 36 | 0.699 | 121 ± 38 | 116 ± 38 | 115 ± 41 | 0.293 |
| HDL cholesterol (mg/dl) | 53 ± 16 | 54 ± 16 | 55 ± 20 | 0.416 | 55 ± 16 | 55 ± 16 | 57 ± 21 | 0.706 | 47 ± 14 | 50 ± 14 | 52 ± 16 | 0.047 |
| Triglycerides (mg/dl) | 154 ± 102 | 145 ± 90 | 143 ± 95 | 0.110 | 144 ± 94 | 140 ± 86 | 137 ± 86 | 0.599 | 190 ± 123 | 159 ± 99 | 157 ± 113 | 0.003 |
| Use of statins (%) | 49.1 | 43.1 | 44.7 | 0.054 | 45.8 | 41.4 | 39.7 | 0.092 | 61.7 | 48.0 | 55.6 | 0.109 |
| Insulin (μU/ml) | 12.2 ± 11.6 | 12.8 ± 34.8 | 11.5 ± 9.5 | 0.546 | 10.8 ± 10.1 | 9.9 ± 8.2 | 9.3 ± 5.7 | 0.180 | 17.3 ± 14.8 | 16.1 ± 13.2 | 15.9 ± 13.4 | 0.561 |
| Glucose (mmol/l) | 5.9 ± 1.7 | 6.2 ± 2.0 | 6.6 ± 2.7 | <0.001 | 5.4 ± 0.7 | 5.4 ± 0.7 | 5.5 ± 1.1 | 0.364 | 8.1 ± 2.5 | 8.4 ± 2.6 | 9.0 ± 3.5 | 0.195 |
| HOMA IR | 3.5 ± 4.7 | 3.4 ± 3.7 | 3.8 ± 5.2 | 0.943 | 2.7 ± 3.4 | 2.4 ± 2.3 | 2.2 ± 1.5 | 0.214 | 6.5 ± 7.9 | 5.9 ± 5.4 | 6.8 ± 7.9 | 0.895 |
| HOMA BCF | 116 ± 95 | 104 ± 83 | 100 ± 87 | 0.002 | 122 ± 94 | 112 ± 84 | 108 ± 88 | 0.201 | 93 ± 93 | 81 ± 76 | 84 ± 101 | 0.125 |
| HbA1c (%) | 6.1 ± 0.9 | 6.2 ± 1.0 | 6.4 ± 1.4 | <0.001 | 5.7 ± 0.4 | 5.7 ± 0.4 | 5.7 ± 0.4 | 0.662 | 7.2 ± 1.3 | 7.2 ± 1.2 | 7.7 ± 1.7 | 0.087 |
| T2DM (%) | 20.6 | 25.6 | 31.8 | 0.001 |  |  |  |  |  |  |  |  |
