# Supplementary material for: Single Nucleotide Polymorphisms of TCF7L2 Are Linked to Diabetic Coronary Atherosclerosis
Source: PLoS One. 2011 Mar 15;6(3):e17978. doi: 10.1371/journal.pone.0017978 (PMC3058059; doi:10.1371/journal.pone.0017978)
Supplement: Table S3 — Subject characteristics with respect to genotypes of rs12255372. Differences in categorical variables were tested for statistical significance with the Chi-square test. For continuous variables ANOVA was applied. Non- normally distributed variables [i.e. age, BMI, HDL cholesterol, LDL cholesterol, triglycerides, fasting insulin, fasting glucose, homeostasis model assessment (HOMA) insulin resistance (IR), HOMA beta cell function (BCF), and haemoglobin A1c (HbA1c)] were log-transformed prior to statistical analysis. Continuous variables are given as mean ± SD (of non log-transformed values). (DOC) [file pone.0017978.s003.doc]

|  | Total cohort | | | | No T2DM | | | | T2DM | | | |
| --- | --- | --- | --- | --- | --- | --- | --- | --- | --- | --- | --- | --- |
| rs12255372 (G>T) | GG | GT | TT | P value | GG | GT | TT | P value | GG | GT | TT | P value |
| Individuals (n) | 832 | 662 | 151 | - | 659 | 494 | 100 | - | 173 | 168 | 51 | - |
| Age (years) | 63.6 ± 10.6 | 64.6 ± 10.5 | 64.7 ± 11.0 | 0.145 | 63.3 ± 10.7 | 64.2 ± 10.6 | 64.0 ± 11.9 | 0.333 | 64.8 ± 10.0 | 65.9 ± 10.0 | 66.0 ± 9.1 | 0.542 |
| Male sex (%) | 67.8 | 67.1 | 57.0 | 0.045 | 66.6 | 67.4 | 58.0 | 0.316 | 72.3 | 66.1 | 54.9 | 0.021 |
| BMI (kg/m^2^) | 27.7 ± 4.4 | 27.4 ± 4.1 | 27.5 ± 4.8 | 0.573 | 27.2 ± 4.1 | 27.0 ± 4.0 | 26.9 ± 4.2 | 0.675 | 29.6 ± 5.0 | 28.7 ± 4.3 | 28.8 ± 5.7 | 0.176 |
| Hypertension (%) | 52.6 | 54.7 | 52.7 | 0.661 | 50.8 | 53.8 | 51.0 | 0.538 | 59.4 | 57.0 | 56.0 | 0.601 |
| Smoking (%) | 59.9 | 60.1 | 51.0 | 0.162 | 57.4 | 58.5 | 48.0 | 0.324 | 69.4 | 64.9 | 56.9 | 0.098 |
| Total cholesterol (mg/dl) | 206 ± 46 | 203 ± 46 | 204 ± 49 | 0.424 | 209 ± 38 | 207 ± 45 | 210 ± 44 | 0.745 | 193 ± 45 | 190 ± 47 | 191 ± 55 | 0.773 |
| LDL cholesterol (mg/dl) | 130 ± 38 | 128 ± 39 | 126 ± 39 | 0.435 | 132 ± 38 | 132 ± 38 | 131 ± 36 | 0.995 | 119 ± 37 | 117 ± 38 | 115 ± 42 | 0.550 |
| HDL cholesterol (mg/dl) | 53 ± 16 | 54 ± 15 | 56 ± 20 | 0.078 | 54 ± 16 | 55 ± 16 | 57 ± 22 | 0.306 | 47 ± 14 | 50 ± 13 | 53 ± 17 | 0.007 |
| Triglycerides (mg/dl) | 154 ± 103 | 144 ± 90 | 140 ± 89 | 0.045 | 145 ± 93 | 139 ± 87 | 138 ± 89 | 0.282 | 188 ± 128 | 162 ± 99 | 146 ± 89 | 0.006 |
| Use of statins (%) | 48.0 | 43.8 | 45.7 | 0.220 | 45.2 | 41.1 | 42.0 | 0.219 | 41.6 | 48.2 | 47.1 | 0.297 |
| Insulin (μU/ml) | 12.2 ± 11.6 | 11.6 ± 9.9 | 10.8 ± 9.2 | 0.180 | 10.8 ± 9.9 | 9.9 ± 8.3 | 9.1 ± 5.8 | 0.082 | 17.6 ± 15.5 | 16.5 ± 12.3 | 13.8 ± 12.9 | 0.289 |
| Glucose (mmol/l) | 5.9 ± 1.7 | 6.2 ± 2.0 | 6.7 ± 2.7 | <0.001 | 5.4 ± 0.7 | 5.4 ± 0.7 | 5.5 ± 1.1 | 0.282 | 8.1 ± 2.4 | 8.5 ± 2.8 | 8.8 ± 3.4 | 0.118 |
| HOMA IR | 3.5 ± 5.0 | 3.4 ± 3.6 | 3.5 ± 5.0 | 0.885 | 2.7 ± 3.3 | 2.4 ± 2.3 | 2.2 ± 1.6 | 0.094 | 6.6 ± 8.1 | 6.1 ± 5.1 | 5.9 ± 7.7 | 0.475 |
| HOMA BCF | 116 ± 96 | 104 ± 83 | 91 ± 75 | <0.001 | 122 ± 96 | 111 ± 82 | 106 ± 75 | 0.156 | 93 ± 94 | 85 ± 84 | 65 ± 66 | 0.021 |
| HbA1c (%) | 6.0 ± 0.9 | 6.1 ± 1.0 | 6.4 ± 1.4 | <0.001 | 5.7 ± 0.4 | 5.7 ± 0.4 | 5.8 ± 0.4 | 0.588 | 7.2 ± 1.3 | 7.3 ± 1.3 | 7.6 ± 1.8 | 0.106 |
| T2DM (%) | 20.8 | 25.4 | 33.8 | <0.001 | - | - | - | - | - | - | - | - |
