# Supplementary material for: Single Nucleotide Polymorphisms of TCF7L2 Are Linked to Diabetic Coronary Atherosclerosis
Source: PLoS One. 2011 Mar 15;6(3):e17978. doi: 10.1371/journal.pone.0017978 (PMC3058059; doi:10.1371/journal.pone.0017978)
Supplement: Table S4 — Subject characteristics with respect to genotypes of rs11196205. Differences in categorical variables study were tested for statistical significance with the Chi-square test. For continuous variables ANOVA was applied. Non- normally distributed variables [i.e. age, BMI, HDL cholesterol, LDL cholesterol, triglycerides, fasting insulin, fasting glucose, homeostasis model assessment (HOMA) insulin resistance (IR), HOMA beta cell function (BCF), and haemoglobin A1c (HbA1c)] were log-transformed prior to statistical analysis. Continuous variables are given as mean ± SD (of non log-transformed values). (DOC) [file pone.0017978.s004.doc]

|  | Total cohort | | | | No T2DM | | | | T2DM | | | |
| --- | --- | --- | --- | --- | --- | --- | --- | --- | --- | --- | --- | --- |
| rs11196205 (G>C) | GG | GC | CC | P value | GG | GC | CC | P value | GG | GC | CC | P value |
| Individuals (n) | 473 | 816 | 355 | - | 377 | 611 | 264 | - | 96 | 205 | 91 | - |
| Age (years) | 63.8 ± 10.6 | 64.5 ± 10.5 | 63.7 ± 10.9 | 0.382 | 63.3 ± 10.8 | 64.2 ± 10.6 | 63.2 ± 11.3 | 0.297 | 66.0 ± 9.6 | 65.3 ± 10.0 | 65.0 ± 9.8 | 0.765 |
| Male sex (%) | 67.4 | 66.4 | 65.4 | 0.527 | 66.6 | 66.4 | 66.5 | 0.795 | 70.8 | 63.3 | 64.8 | 0.380 |
| BMI (kg/m^2^) | 27.6 ± 4.6 | 27.6 ± 4.2 | 27.4 ± 4.2 | 0.821 | 27.0 ± 4.2 | 27.1 ± 4.1 | 27.0 ± 3.8 | 0.876 | 29.8 ± 5.5 | 29.0 ± 4.3 | 28.5 ± 5.1 | 0.184 |
| Hypertension (%) | 50.0 | 56.3 | 51.5 | 0.514 | 48.1 | 54.9 | 50.8 | 0.362 | 57.3 | 60.2 | 53.4 | 0.615 |
| Smoking (%) | 59.4 | 59.9 | 57.2 | 0.562 | 58.1 | 57.6 | 54.9 | 0.451 | 64.6 | 66.8 | 63.7 | 0.912 |
| Total cholesterol (mg/dl) | 207 ± 48 | 204 ± 45 | 203 ± 46 | 0.429 | 210 ± 48 | 208 ± 44 | 207 ± 44 | 0.780 | 195 ± 46 | 191 ± 47 | 190 ± 50 | 0.738 |
| LDL cholesterol (mg/dl) | 130 ± 40 | 129 ± 38 | 127 ± 39 | 0.639 | 132 ± 40 | 132 ± 37 | 131 ± 38 | 0.836 | 121 ± 39 | 118 ± 38 | 115 ± 40 | 0.526 |
| HDL cholesterol (mg/dl) | 53 ± 17 | 54 ± 15 | 54 ± 17 | 0.214 | 54 ± 17 | 55 ± 16 | 55 ± 18 | 0.320 | 47 ± 15 | 49 ± 14 | 50 ± 15 | 0.192 |
| Triglycerides (mg/dl) | 157 ± 109 | 145 ± 88 | 147 ± 98 | 0.063 | 148 ± 98 | 138 ± 87 | 142 ± 86 | 0.184 | 193 ± 137 | 165 ± 89 | 162 ± 128 | 0.030 |
| Use of statins (%) | 48.4 | 44.7 | 45.9 | 0.414 | 44.8 | 42.6 | 42.8 | 0.569 | 62.5 | 51.2 | 54.9 | 0.287 |
| Insulin (μU/ml) | 12.5 ± 11.9 | 11.6 ± 10.8 | 11.5 ± 8.9 | 0.896 | 10.6 ± 9.1 | 10.2 ± 10.0 | 10.0 ± 5.6 | 0.924 | 19.4 ± 174 | 15.7 ± 12.1 | 15.5 ± 13.0 | 0.216 |
| Glucose (mmol/l) | 5.9 ± 1.7 | 6.1 ± 2.0 | 6.2 ± 2.2 | 0.065 | 5.4 ± 0.7 | 5.4 ± 0.7 | 5.4 ± 0.9 | 0.771 | 8.0 ± 2.6 | 8.4 ± 2.6 | 8.6 ± 3.0 | 0.322 |
| HOMA IR | 3.6 ± 5.3 | 3.4 ± 4.1 | 3.5 ± 4.2 | 0.925 | 2.6 ± 2.5 | 2.6 ± 3.4 | 2.4 ± 1.6 | 0.899 | 7.4 ± 9.8 | 6.1 ± 5.1 | 6.2 ± 6.9 | 0.593 |
| HOMA BCF | 117 ± 104 | 107 ± 83 | 104 ± 81 | 0.171 | 120 ± 103 | 116 ± 86 | 111 ± 74 | 0.911 | 106 ± 108 | 86 ± 84 | 83 ± 95 | 0.029 |
| HbA1c (%) | 6.1 ± 0.9 | 6.1 ± 1.0 | 6.2 ± 1.2 | 0.006 | 5.7 ± 0.4 | 5.7 ± 0.4 | 5.7 ± 0.4 | 0.038 | 7.1 ± 1.3 | 7.3 ± 1.3 | 7.5 ± 1.6 | 0.111 |
| T2DM (%) | 20.8 | 25.4 | 33.8 | 0.058 | - | - | - | - | - | - | - | - |
