# Supplementary material for: Single Nucleotide Polymorphisms of TCF7L2 Are Linked to Diabetic Coronary Atherosclerosis
Source: PLoS One. 2011 Mar 15;6(3):e17978. doi: 10.1371/journal.pone.0017978 (PMC3058059; doi:10.1371/journal.pone.0017978)
Supplement: Table S5 — Estimated frequency of common haplotypes and their associations with significant coronary artery disease. Odds ratios (OR), 95% confidence intervals (CI), and p-values were calculated by chi-squared tests by comparing each haplotype to all remaining haplotypes. (DOC) [file pone.0017978.s005.doc]

|  | Haplotype |  | CAD (-) % | CAD (+) % | OR [95%CI] | P value |
| --- | --- | --- | --- | --- | --- | --- |
| rs7903146 C>T | rs12255372 G>T | rs11196205 G>C |
| C | G | G | 53.6 | 52.2 | 0.94 [0.82-1.09] | 0.417 |
| C | G | C | 14.9 | 13.3 | 0.88 [0.72-1.07] | 0.196 |
| C | T | C | 2.1 | 1.4 | 0.65 [0.38-1.11] | 0.111 |
| T | G | C | 3.7 | 3.4 | 0.91 [0.63-1.33] | 0.637 |
| T | T | C | 25.1 | 29.0 | 1.22 [1.04-1.43] | 0.013 |
